# Using isometric log-ratio in compositional data analysis for developing a groundwater pollution index

**DOI:** 10.1038/s41598-024-63178-6

**Published:** 2024-05-28

**Authors:** Junseop Oh, Kyoung-Ho Kim, Ho-Rim Kim, Sunhwa Park, Seong-Taek Yun

**Affiliations:** 1 Department of Earth and Environmental Sciences, Korea University, Seoul, 02841 South Korea; 2 Korea Environment Institute, Sejong, 30147 South Korea; 3 Korea Institute of Geoscience and Mineral Resources, Daejeon, 34132 South Korea; 4 National Institute of Environmental Research, Incheon, 22689 South Korea

**Keywords:** Groundwater pollution index (GPI), Compositional data analysis (CoDa), Robust principal component analysis (RPCA), Isometric log-ratio (ILR) transformation, Environmental chemistry, Environmental impact

## Abstract

This study introduces a novel groundwater pollution index (GPI) formulated through compositional data analysis (CoDa) and robust principal component analysis (RPCA) to enhance groundwater quality assessment. Using groundwater quality monitoring data from sites impacted by the 2010–2011 foot-and-mouth disease outbreak in South Korea, CoDa uncovers critical hydrochemical differences between leachate-influenced and background groundwater. The GPI was developed by selecting key subcompositional parts (NH_4_^+^-N, Cl^-^, and NO_3_-^-^N) using RPCA, performing the isometric log-ratio (ILR) transformation, and normalizing the results to environmental standards, thereby providing a more precise and accurate assessment of pollution. Validated against government criteria, the GPI has shown its potential as an alternative assessment tool, with its reliability confirmed by receiver operating characteristic curve analysis. This study highlights the essential role of CoDa, especially the ILR -transformation, in overcoming the limitations of traditional statistical methods that often neglect the relative nature of hydrochemical data. Our results emphasize the utility of the GPI in significantly advancing groundwater quality monitoring and management by addressing a methodological gap in the quantitative assessment of groundwater pollution.

## Introduction

Groundwater monitoring is an essential requirement for sustainable water resource management especially in areas where aquifers are under anthropogenic pressures such as overexploitation and pollution^1,2^. Groundwater quality monitoring, especially around hazardous waste storage and disposal facilities, is typically conducted in compliance with environmental regulations (e.g., EPA, 1986) to detect and prevent contamination that threatens human health and the environment^3^. Environmental criteria such as drinking water standards are used to ascertain the impacts and/or risks of groundwater contamination from the groundwater quality monitoring. However, such criteria often serve limited statutory and legal purposes, and may not provide a comprehensive understanding of the present status and temporal trend of groundwater quality for an early warning of groundwater contamination^4^. Therefore, it is needed to measure the relative concentrations of hazardous substances in groundwater compared to their environmental standards and/or background levels^5–7^.

In this context, the development of water quality index (WQI), such as the groundwater pollution index (GPI), serves as a surveillance tool to enhance both surface and groundwater quality assessment^8–11^. WQI and/or GPI are comprehensive metrics that aggregate various hydrochemical and biological parameters into a single, simplified score, providing a concise representation of overall water quality. Globally, a number of WQI models have been proposed, proving helpful for policy development and decision-making in water resource management^12,13^. Among them, multivariate statistical methods, especially dimensionality reduction techniques such as principal component analysis (PCA) and factor analysis (FA), have become prevalent in WQI development^14–17^. For instance, PCA provides an objective method for WQI development, typically using weighted linear combination of multiple parameters to calculate a minimal number of independent indices (scores), preserving the multivariate covariance structure dataset^18–20^. Moreover, even if given groundwater quality data may contain outliers, PCA can involve a robust procedure (e.g. robust PCA)^21,22^. However, traditional statistical approaches fail to adequately account for the compositional nature of groundwater quality datasets, which include multiple hydrochemical parameters. This oversight can lead to inaccurate conclusions, primarily due to two significant limitations: outliers and data closure issues^23,24^. Consequently, to address these limitations and facilitate the robust statistical development of an optimal WQI, the implementation of compositional data analysis (CoDA) is essential.

However, many statistical approaches have failed to consider the relative nature of groundwater quality data with multiple hydrochemical parameters, potentially leading to somewhat erroneous conclusions due to the relative variation of hydrochemical parameters necessitating the use of compositional data analysis (CoDa) for the statistical development of WQI.

Most environmental data involving groundwater quality data are compositional since they are usually reported in a relative unit (e.g., mg/L, mole/L, meq/L, etc.)^25–27^. Compositional data is typically defined as vectors with strictly positive summing up to a constant (i.e., closed number systems)^28^, and recently referred to as being parts of a whole which carry relative information^25,29^. Standard statistical approaches of compositional data are not straightforward due to the fact that they follow the Aitchison geometry (not the Euclidean geometry), pertaining to a sample space called the simplex^28,30,31^. Consequently, CoDA involves complex data transformation from the simplex to the Euclidean space before performing statistical analyses; three types logratio transformations has been suggested: the additive logratio (ALR) and the centered logratio (CLR) transformation^28^, and more recently, the isometric log-ratio (ILR) transformation^32^.

Recently, the increasing use of multivariate CoDa in interpreting groundwater quality data has led to the recommendation of the ILR-transformation before analysis^21,22^. This approach avoids the singularity issue inherent in the CLR transformation and maintains isometry between the simplex and real space^32^. The ILR transformation forms an orthonormal axis system (known as ILR-coordinates or balances) by dividing multivariate variables (compositioanl parts) into non-overlapping subsets through a sequential binary partition (SBP)^30^. Understanding the underlying processes in the data is beneficial for selecting appropriate SBP and forming ILR-coordinates^33^. This approach ensures subcompositional coherence, meaning that each subset of parts (subcomposition) preserves the information of the entire composition^28,34^. Consequently, ILR-coordinates of a selected subcomposition, which explain significant compositional changes in groundwater quality data, enhance the effectiveness of data exploration and interpretation^25,35,36^. Although CoDa, particularly the ILR transformation, is essential for statistical analysis of groundwater quality data, its application in indexing groundwater pollution has not yet been utilized.

This study aims to bridge this gap by investigating the applicability of the CoDa approach in developing a Groundwater Pollution Index (GPI). We propose a straightforward GPI, derived from the ILR-coordinate of a critical subcomposition, to effectively indicate groundwater pollution. Utilizing groundwater quality monitoring data from a study on the impact of livestock mortalities burial on groundwater^37^, we applied CoDa and robust PCA to assess multivariate hydrochemical data. This approach minimized the impact of outliers and accurately represented important subcompositional parts (parameters). From this analysis, we suggested a simple GPI as the ILR-coordinate of a selected important subcomposition, effectively revealing leachate pollution in groundwater. Our research addresses the current methodological gap by focusing on the indexing of groundwater pollution through compositional data analysis, marking a crucial step towards more accurate and reliable groundwater quality assessment.

## Materials and methods

### Groundwater quality monitoring data

The burial pit sites (n = 30), from which our groundwater quality monitoring data were sourced, are regionally distributed across Korea. They were strategically selected to cover a broad geographic area and to provide a comprehensive dataset, reflecting the diverse hydrogeological conditions of the region. This study was based on the groundwater quality monitoring data collected from a monitoring program conducted to investigate leachate leakages from livestock carcass burial pits formed during the 2010–2011 foot-and-mouth disease (FMD) outbreak in South Korea. This monitoring program was carried out by the National Institute of Environmental Research (NIER) of South Korea on a quarterly basis throughout 2012 for 270 burial pit sites. As the monitoring result, the leachate leakages were confirmed in 29.6% of the burial pits according to the government (Ministry of Environment: ME) guideline for environmental management of carcass burial pits that involves the environmental criterion for three water quality parameters of electric conductivity (EC > 800 mS/cm), NH_4_^+^-N (> 10 mg/L), and Cl^−^ (> 100 mg/L)^37,38^. Details about the burial sites established following the FMD epidemic and the subsequent monitoring programs are described in previous studies^37,39^.

For our analysis, we used the groundwater quality monitoring data only collected from 30 burial pit sites involving multiple parameters: 3 in-situ measurements (pH, EC, ORP), 10 hydrochemical ions (DO, BOD, COD, Total N, NH_4_^+^-N, Cl^-^, Ca^2^^+^, Na^+^, TP and PO_4_^3^), and 2 microbial parameters (TB and TC). This monitoring data includes a total of 100 analytical results (samples) comprising two types of groundwater samples representing livestock carcass leachate and nearby groundwater compositions, respectively. The leachate samples were obtained from the perforated drainpipes (as leachate wells: LW) installed at the top of burial pits, while the groundwater samples were collected from monitoring wells (MW) installed 10 m downgradient from burial pits. The nonparametric Mann–Whitney U test was used to evaluate the differences of groundwater pollution indices as well as water quality parameters between the two groups (LW and MW). The water quality analyses and measurements for the parameters were conducted at NIER following the standard methods for drinking water in South Korea^40^; a detailed description of the methods (including QA/QC) can be found the original publication of NIER (2012)^41^.

Note that the groundwater quality monitoring data was selected because it clearly demonstrates the relative compositional changes between leachate-influenced and background groundwater. This distinction is crucial for validating the applicability of the ILR in developing a robust GPI within the framework of multivariate CoDA. To quantify groundwater contamination (i.e., leachate leakage from burial pits) using the proposed GPI, an assessment was conducted across the entire dataset. This involved evaluating the differences in contamination indices between two groundwater groups (Leachate Wells, LW, and Groundwater Wells, GW) and examining the discriminatory ability of the contamination index.

### Compositional data analysis (CoDA)

#### Log-ratio transformation

The groundwater quality monitoring data covers the compositional variables (i.e., hydrochemcial parameters) except for some physicochemical parameters and total coliforms. As mentioned above, compositional data is defined as vectors of positive real numbers in which the components (or parts) carry only relative information of some whole data^28^. Since compositional data are constrained to a sample space, called simplex, the standard statistical analyses relying on the Euclidean geometry may obtain spurious results^28,30,31^. Consequently, compositional data analysis (CoDA) involves complex data transformation from the simplex to the Euclidean space before performing statistical analyses; three types log-ratio transformations has been suggested: the Additive Log-Ratio (ALR) and the Centered Log-Ratio (CLR) transformation^28^, and more recently, the Isometric Log-Ratio (ILR) transformation^32^.

The ILR-transformation maps the D-parts (D-dimensional variables) of the composition in the simplex (S^D^) into D-1 ILR-coordinates (called balances) in the Euclidean space (R^D-1^), allowing for standard statistical analysis techniques to be applied and preserving the relative information between parts^32,34^. The ILR-coordinates are defined using an orthonormal basis, which is created through a process called sequential binary partition (SBP)^30,42^. This procedure divides the parts of a full composition (or subcomposition) into binary non-overlapping groups in a sequential and hierarchical manner until all of the groups have only a single part. Given a composition of D parts, the ILR (z_i_) between two non-overlapping groups can be defined for each of the SBP steps (D-1) as follows:

1 $$ ilr \left({z}_{i}\right)=\sqrt{\frac{{r}_{i}\times {s}_{i}}{{r}_{i}+{s}_{i}}}ln\frac{g({c}_{+})}{g({c}_{-})} with i=1,\dots ,D-1,$$

where g(c_+_) represents the geometric mean of the *r* variables of the numerator of the balance, and g(c_-_) represents the geometric mean of the *s* variables of the denominator. The ILR-coordinates, especially in high dimensions, are not readily interpretable due to the lack of a direct one-to-one relationship between raw and transformed parts. This necessitates expert-knowledge (e.g., geochemical stoichiometry to construct informative balances^36^. On the other hand, the CLR-transformation preserve the D-parts of the composition and useful for examining the relative variation of each part with respect to the whole compositional data. The CLR from S^D^ to R^D^ is defined as

2 $$ clr \left(x\right)=\left[ln\frac{{x}_{1}}{g\left(x\right)},\dots ,ln\frac{{x}_{D}}{g\left(x\right)}\right],$$

where g(x) is the geometric mean of the composition x. However, the CLR-transformed coordinates are sub-compositionally incoherent since it depends on which parts are included in the geometric means as a common divisor. Additionally, their covariance and correlation matrices are singular due to the inherent constraint of all coordinates summing to zero^28,32^. Thus, ILR-transformation is highly recommended prior to the multivariate compositional data analysis^21,22^. Here, we employed the CLR-coordinates to represent the results (loadings and scores) from robust PCA (RPCA) built on ILR-transformed data, back-transformed to CLR space. This facilitates the development of more informative ILR balances as a groundwater pollution index for delineating leachate-induced groundwater pollution.

#### Robust principal component analysis (RPCA)

Principal Component Analysis (PCA) has been widely used to assess groundwater quality and to identify underlying processes such as groundwater contamination^43–46^. Moreover, it serves as an objective method for calculating water quality indices (WQI) through weighted linear combinations by selecting and weighting important groundwater quality parameters^8^. Robust principal component analysis (RPCA) is an approach robust to outlying samples unlike classical PCA which is sensitive to outliers^47^. However, PCA is sensitive to outliers in groundwater quality data, which typically exhibit a skewed covariance structure (i.e., a non-multivariate normal distribution)^48^. RPCA is an approach that is robust to outlying samples by identifying and mitigating the influence of outliers, thus providing a more reliable interpretation of the underlying processes in compositional data compared to traditional PCA^21,47^.

RPCA uses the minimum covariance determinant (MCD) estimator to calculate the sample arithmetic mean vector and the sample covariance matrix used for performing PCA. The MCD is designed to search a subset of at least h observations (> half of the total sample size n) with the smallest determinant of their sample covariance matrix resistant to outliers (for details, see Rousseeuw and Driessen, 1999)^49^. Therefore, RPCA based on MCD determines the location (mean) and scatter (covariance matrix) of data with a multivariate normal distribution, and the associated eigenvectors and eigenvalues provide the PC loadings and scores robust to outliers.

RPCA has been effectively applied in compositional data analysis^50–52^. The ILR transformation is essential for RPCA preferred over the CLR, due to the singularity of the CLR's covariance matrix, which results from the constant sum constraint of its components^52^. In this study, RPCA was conducted to establish the optimal SBP for a comprehensive explanation of groundwater quality monitoring data. This involved the identification of a specific subcomposition aimed at evaluating the influence of leachate from livestock carcasses on groundwater, for which the relevant ILR-coordinates were proposed. The ILR transformed data based on an arbitrary orthonormal basis were used in RPCA. For enhanced interpretability, the results (loadings and scores) of RPCA were back-transformed to the CLR and visually depicted via a biplot.

#### ILR-based Groundwater pollution index (GPI)

This study introduces a novel approach for constructing a GPI through the ILR transformation of multivariate hydrochemical parameters in groundwater quality monitoring data. This method involves a series of statistical procedures, incorporating PCA and CoDa. The framework for developing the ILR-based GPI consists of three major steps: (i) selecting key subcompositional parts using PCA (robust PCA in this study), (ii) performing the ILR transformation via Sequential Binary Partitioning (SBP), and (iii) carrying out normalization according to existing environmental standards or government guidelines. Detailed explanations of these steps are as follows.

PCA aids in selecting a subcomposition of key parameters (parts) that delineate the relative compositional change indicative of groundwater pollution (i.e., the leachate impact on background groundwater quality). These selected subcompositional parts are then transformed into ILR-coordinates (balances) via the SBP. Subsequently, the first ILR-coordinate with the highest variance is chosen to be used as a univariate GPI, effectively contrasting leachate-influenced parameters against those prevalent in the background groundwater. For instance, when dealing with a subcomposition of two parts, the ILR-coordinate Z is defined as follows, according to Eq. (1):

3 $$ Z=\frac{1}{\sqrt{2}}ln\frac{{C}_{P}}{{C}_{B}}$$

where C_P_ and C_B_ represent the concentrations in mg/L of parameters indicative of pollution and background respectively.

We normalized the ILR-coordinate Z proposed as a univariate GPI using the environmental criteria mentioned in Sect. 2.1. This procedure also validates the ILR-based GPI for practical purposes by comparing it with the government guidelines. The validation compares outcomes categorized into binary groups (polluted and non-polluted), determined by different cutoff values along the ILR-coordinate, with those classified according to the environmental criteria. We calculate diagnostic measures such as sensitivity (true positive rate) and specificity (true negative rate) to assess classification performance at varying cutoff points. From these results, an optimal cutoff of the ILR-based GPI, which yields the most similar classification result to the environmental criteria, can be derived. The optimal cutoff is identified using a receiver operating characteristic (ROC) curve, which plots sensitivity against 1-specificity at various cutoff points. It is chosen at the point on the ROC curve where both sensitivity and specificity reach their highest values.

Finally, the ILR-coordinate *Z* can be scaled to a centered value *Z'* by subtracting the cutoff and normalized to a range of 0 to 1 using the maximum and minimum values as follows:

4 $$ GPI=\frac{Z-\text{min}({Z}{\prime})}{\text{max}\left({Z}{\prime}\right)-\text{min}({Z}{\prime})}$$

This results in a normalized GPI, ranging from 0 to 1, which probabilistically assesses the impact of leachate on groundwater. Here, a normalized GPI value exceeding 0.5 indicates leachate pollution in accordance with the environmental criteria.

Our approach in alignment with the principle of subcompositional coherence in CoDa^28^. This principle ensures that an analysis conducted on a subcomposition is consistent with the analysis of the entire composition. This method provides a statistically reliable GPI that carries the relative information in groundwater quality monitoring data, transforming it from simplex to Euclidean space via the the log-ratio transformation.

All statistical procedures (i.e., CoDa and RPCA) of this study was carried out using the robCompositions^53^ package in R software^54^.

## Results and discussion

### Characteristics of groundwater quality monitoring data: absolute versus relative concentrations

Table 1 presents that the median values of most groundwater quality parameters except for redox potential (ORP) have significantly higher (*p* < 0.05) concentrations in the leachate (LW) than in the nearby groundwater (MW). Previous studies have shown that such livestock mortality leachate contains high concentrations of inorganic and organic compounds (e.g., ammonium, alkalinity, chloride, sulfate, BOD, and COD) as a result of carcass decomposition^37,55,56^. The lower ORP in the leachate (median = -66 mv) compared to the surrounding groundwater (median = 115 mv) is due to anaerobic conditions prevailing in the burial pits^44,57,58^. The carcass leachate leakage from burial pits thus induces the subsequent increases of ionic concentrations in groundwater, exhibiting positive correlations with EC and TDS concentrations but negative correlations with ORP.

**Table 1:** Statistical summary of groundwater quality data (n = 420) collected from leachate wells (LW) and groundwater monitoring wells (MW) in the livestock carcass burial pits (n = 30).

Category	Quantile	pH	EC (µS/cm)	ORP (mV)	DO (mg/L)	BOD (mg/L)	COD (mg/L)	TN (mg/L)	NH_4_^+^-N (mg/L)	NO_3_^—^N (mg/L)	Cl^-^ (mg/L)	Ca^2+^ (mg/L)	Na^+^ (mg/L)	TP (mg/L)	PO_4_^3-^ (mg/L)
Total Samples (n = 480)	Q25	6.20	304.5	6.0	1.3	1.3	13.8	7.3	0.0	1.3	17.9	21.4	11.0	0.0	0.0
	Q50	6.58	599.5	93.8	2.7	4.8	40.0	20.9	0.9	2.9	36.4	41.5	19.1	0.2	0.0
	Q75	6.94	1342.5	160.5	4.2	31.4	568.3	86.3	28.5	7.7	104.9	101.0	50.5	1.6	0.0
Leachate Wells (n = 75)	Q25	6.00	2497.0	− 197.0	0.7	451.0	4120.0	735.8	324.4	0.6	123.2	98.4	63.1	1.1	0.0
	Q50	6.41	4930.0	− 66.0	1.5	1879.2	11,333.3	2890.9	1554.0	5.0	279.3	249.3	185.9	5.2	0.3
	Q75	6.83	8790.0	62.0	3.0	6765.1	44,000.0	7184.7	3666.0	13.5	723.4	582.9	591.0	14.0	1.0
Monitoring Wells (n = 345)	Q25	6.23	279.0	34.0	1.4	0.9	11.0	5.8	0.0	1.3	16.0	20.0	10.1	0.0	0.0
	Q50	6.61	456.0	115.0	3.0	2.7	24.0	13.9	0.3	2.8	27.3	35.0	15.6	0.1	0.0
	Q75	6.95	854.0	173.0	4.6	8.9	105.0	33.8	3.8	6.9	57.6	71.6	27.9	0.6	0.0
*p*-value		*p* < .05	*p* < .001	*p* < .001	*p* < .001	*p* < .001	*p* < .001	*p* < .001	*p* < .001	*p* < .01	*p* < .001	*p* < .001	*p* < .001	*p* < .001	*p* < .001

**Figure 1:**
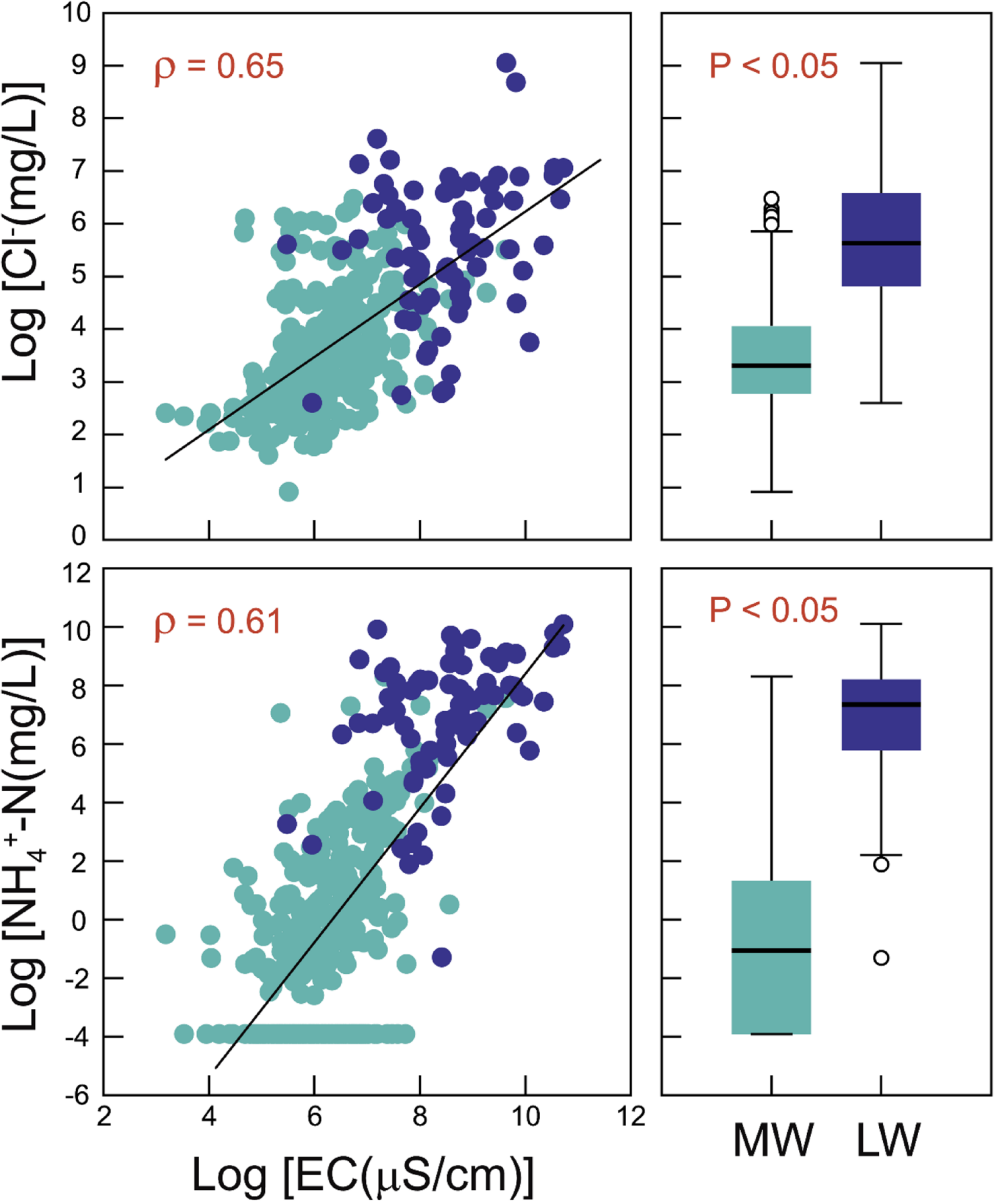
Bivariate relationships between log-transformed concentrations of Cl^-^ and NH_4_^+^-N ions and log-transformed electrical conductivity (EC) (left), and the comparison of these two log-scaled concentrations between monitoring wells (MW) and leachate wells (LW) (right).

Given the elevated ionic concentrations within the leachate wells (LW) relative to the adjacent groundwater monitoring wells (MW), the log-scaled concentrations of Cl- and NH_4_^+^-N ions have strong positive Spearman correlations with EC values (ρ = 0.65 and 0.61), respectively in the total dataset (combined LW and MW) (Fig. 1). This indicates that the influence of leachate infiltration on proximate groundwater can be quantitatively diagnosed by measuring the correlation coefficients among hydrochemical parameters in the groundwater quality monitoring data. Nevertheless, as mentioned above (Sect. 2.2), the hydrochemical parameters are inherently compositional parts that carry relative information. Therefore, the correlations computed between any pair of log-transformed variables can be spurious and the log-ratio transformations such as centered log-ratio (CLR) and isometric log-ratio (ILR) are necessary for hydrochemical parameters^28,32^.

**Figure 2:**
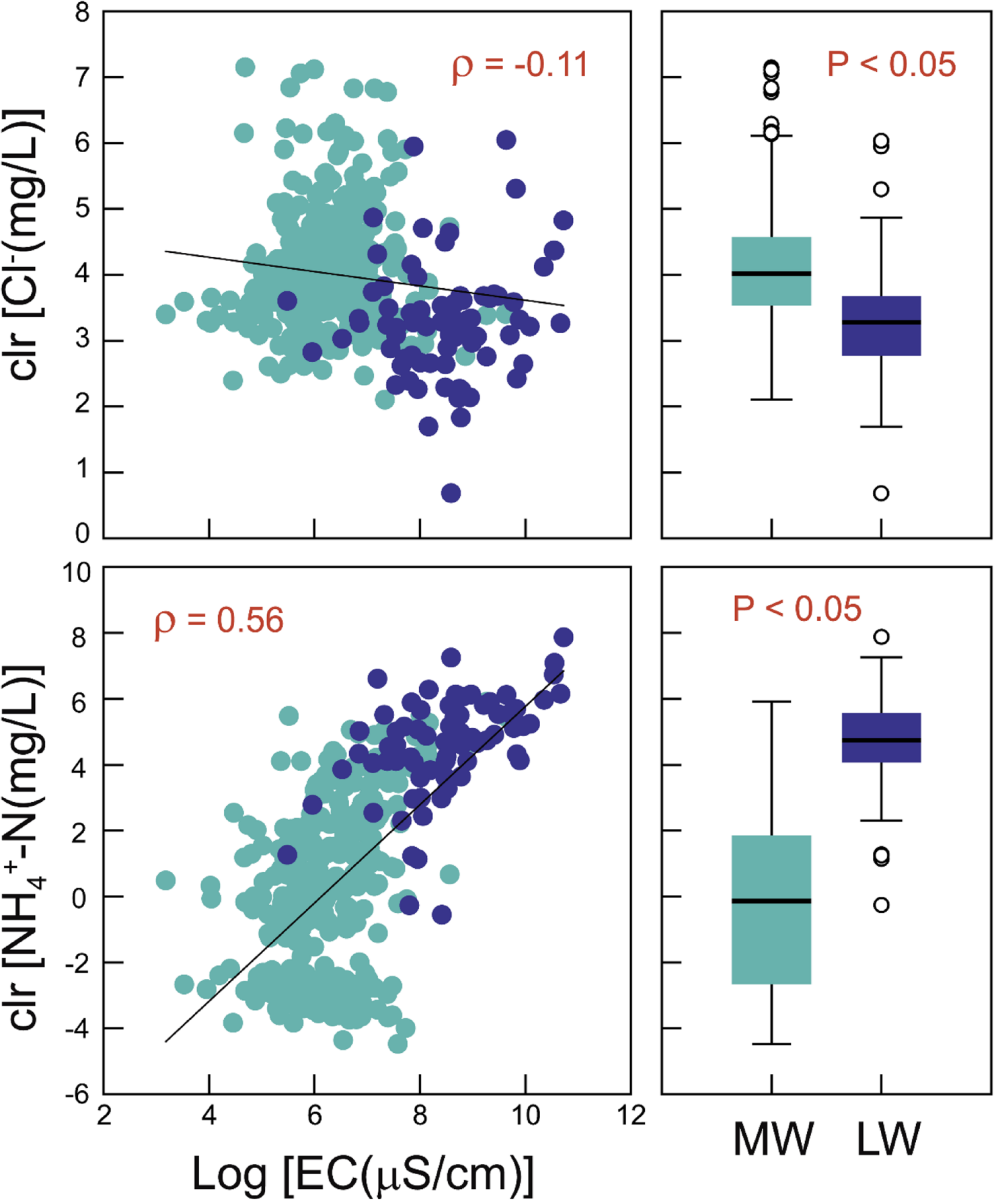
Bivariate relationships between clr-transformed concentrations of Cl^-^ and NH_4_^+^-N ions and log-transformed electrical conductivity (EC) (left), and the comparison of these two clr values between Monitoring Wells (MW) and Leachate Wells (LW) (right).

Figure 2 shows the bivariate relationships with correlation coefficients between the CLR-transformed values (i.e., relative to the geometric mean of all components) of Cl^-^ and NH_4_^+^-N and log-transformed EC. A positive correlation is observed for NH_4_^+^-N (ρ = 0.56), consistent with the log-transformed data. Conversely, Cl^-^ reveals a negative correlation (ρ = − 0.11) despite a positive association in its log-transformed data. This is attributed to the relatively high Cl^-^ concentration in the groundwater from monitoring wells (MW) compared to that in the leachate (LW) (right in Fig. 2). The elevated Cl^-^ levels in MW result from the influence of agricultural practices, such as the use of livestock manures and fertilizers, which affect the background levels of the groundwater near the burial pits, unlike NH_4_^+^-N, which is primarily originated from carcass leachate. These results demonstrate that the correlation structure in the total dataset can change when considering the relative compositions of individual hydrochemical parameters.

### Assessing the influence of leachate on groundwater quality using multivariate CoDa and RPCA

**Figure 3:**
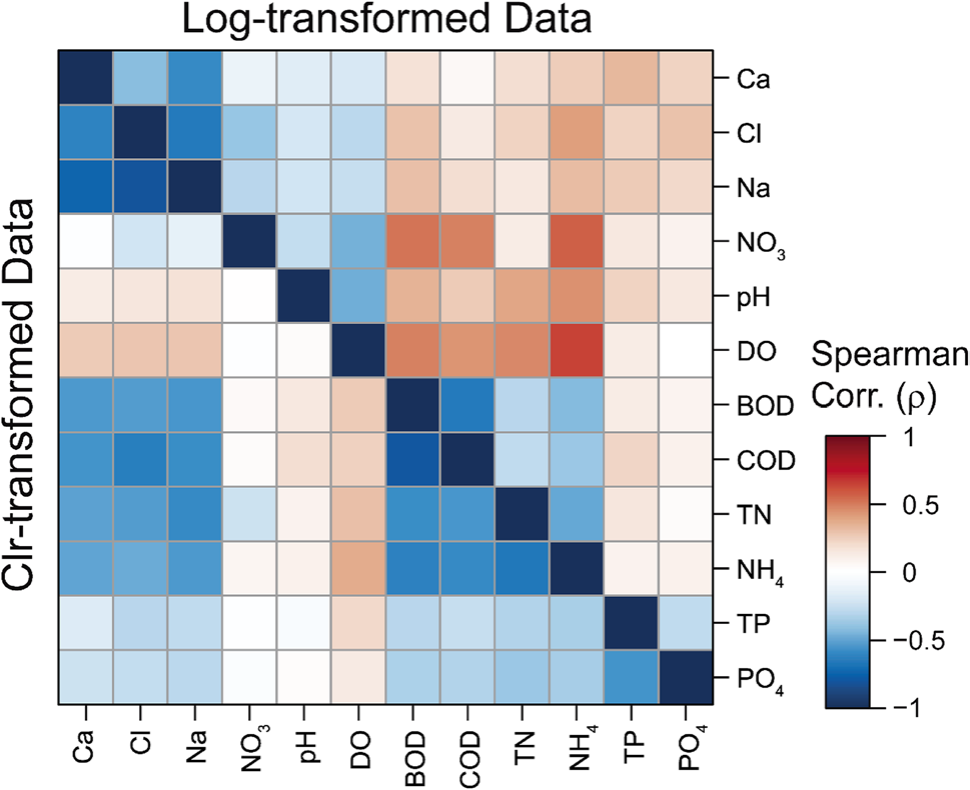
Correlation matrix of twelve hydrochemical parameters in the total dataset, showing Spearman correlation coefficients for log-transformed data (upper triangle) and for clr-transformed data (lower triangle).

Given the multivariate compositional nature of hydrochemical data, it is necessary to employ a correlation matrix derived from log-ratio transformations to examine the interrelationships among various compositional parameters. Figure 3 shows the significant differences between between the correlation matrices of log-transformed and CLR-transformed variables (excluding EC, ORP, total bacteria, and total coliform) in the total dataset. This comparison demonstrates that the type of data transformation significantly influences the outcome of correlation analysis, as previously shown (Figs. 2 and 3). The result of log-transformed data (lower section of Fig. 3) reveals that the nine hydrochemical parameters (Cl^-^, Ca^2+^, Na^+^, BOD, COD, Total N, NH_4_^+^-N, Total P, PO_4_^3-^), predominantly concentrated in the leachate (LW), exhibit positive correlations with each other. In contrast, these parameters display negative correlations with pH and redox-sensitive parameters (DO and NO_3_^-^-N), which typically decrease under anaerobic conditions.

On the other hand, the correlation matrix for CLR-transformed data (upper section of Fig. 3) explains the relative compositional relationships based on their source attributions. For instance, parameters primarily originating from leachate (BOD, COD, Total N, NH_4_^+^-N) show inverse relationships with those dominant in background groundwater (Cl^-^, Ca^2+^, Na^+^) as well as with redox-sensitive ions (DO and NO_3_^-^-N). It is noteworthy that the relative compositions of hydrochemical data are inherently influenced by the proportional contributions from various solute sources, such as carcass leachate and agricultural practices (e.g., livestock manures and fertilizers). Therefore, the application of CoDa (i.e., log-ratio transformations) can be more useful and relevant than using absolute concentrations (such as raw or log-transformed data) for a statistical and practical assessment of the impact of leachate leakage on groundwater quality.

**Figure 4:**
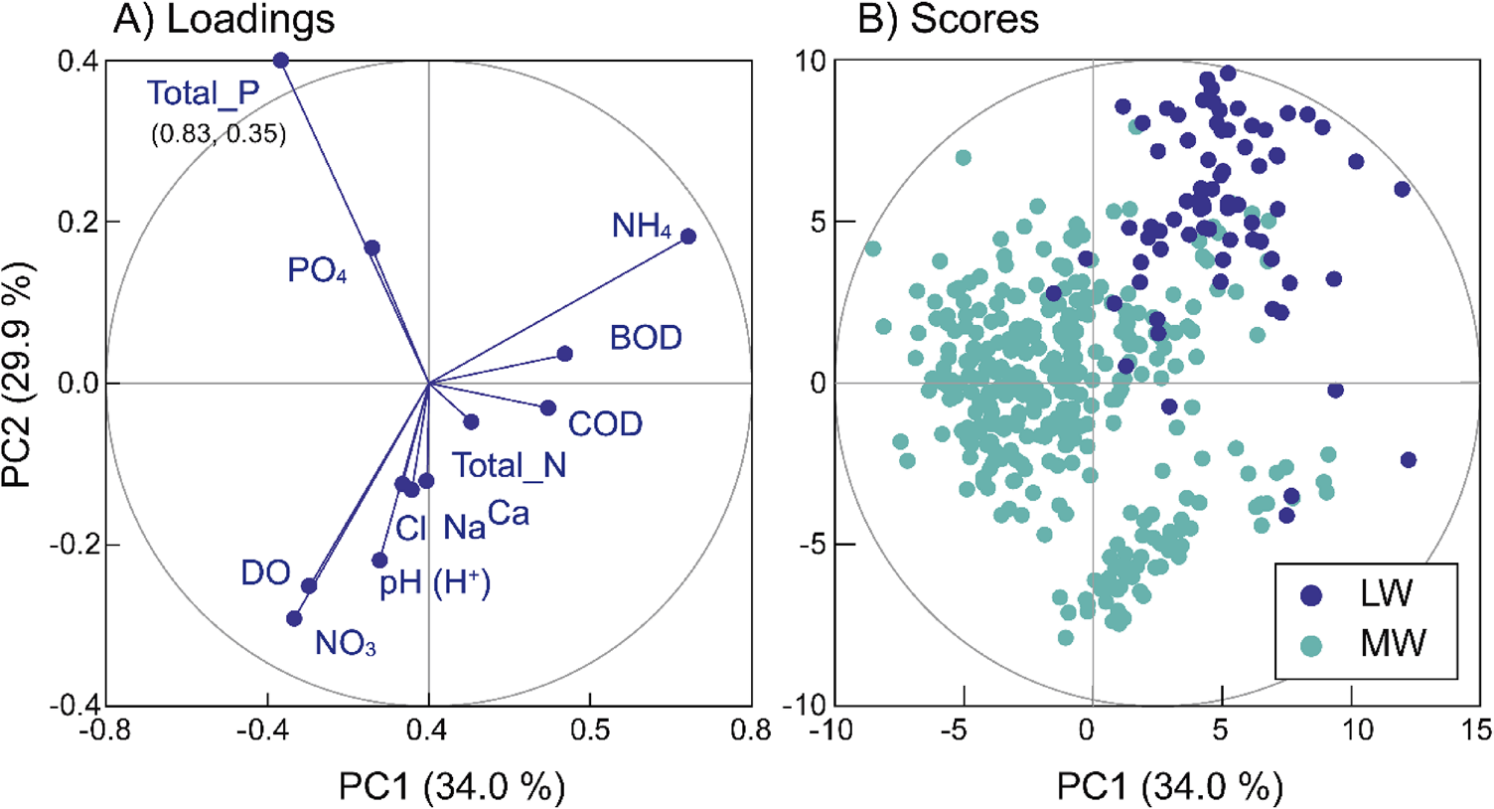
Biplots of loadings and scores from robust principal component analysis (RPCA) of hydrochemical Parameters using isometric log-ratio (ilr). The illustrated loadings and scores have been back-transformed into centered log-ratio (clr) values for interpretation.

In the context of multivariate CoDa, RPCA provides a more comprehensive explanation of the relative compositional changes in hydrochemical data. In this study, RPCA was applied to the ILR-transformed data, and then the loadings and scores of RPCA were back-transformed to the CLR-coordinates. From the result, the first two principal components (PC1 and PC2), accounting for 34.0 and 29.9% of the total variance, are extracted from the ILR-transformed data (Table 2 and Fig. 4). The loadings exhibit a correlation (or covariance) structure among the twelve hydrochemical parameters. Notably, PC1 has positive correlations with NH_4_^+^, BOD, and COD, which are predominantly enriched in carcass leachate, while it shows negative correlations with redox-sensitive parameters such as DO and NO_3_^-^-N. Ions such as Cl^-^, Na^+^, and Ca^2+^, despite their high absolute concentrations in leachate, show only weak correlations with PC1. This is attributed to their relative abundance in the background groundwater. On the other hand, PC2 has correlations with total P and PO_4_^3-^. However, both variables are redundant in the interpretation since they are immobile in groundwater due to their adsorption on soils and sediments^59^. Therefore, PC1 delineates the impact of leachate on groundwater quality, showing the relative increase in ionic concentrations compared to background levels and the formation of anaerobic conditions. These results are identical to the outcomes obtained from the correlation analysis on the CLR-transformed data.

**Table 2:** Isometric log-ratio (ilr) of five selected subcompositions and their corresponding binary partitions, indicating the impact of leachate on groundwater. The table also includes Spearman correlation coefficients (R) between the ilr and the PC1 score from RPCA.

Isometric-log ratio (ilr)	Selected Parts (dimension)	Binary partition of selected balance (ilr)		ρ
		Numerator ( +)	Denominator (-)	
Z_1_	12	BOD, COD, NH_4_^+^-N	DO, H^+^, NO_3_-N, TN, Cl^-^, Ca^2+^, Na^+^	0.75
Z_2_	7	BOD, COD, NH_4_^+^-N	NO_3_-N, Cl^-^, Na^+^, DO	0.83
Z_3_	5	BOD, COD, NH_4_^+^-N	NO_3_-N, Cl^-^	0.81
Z_4_	5	NH_4_^+^-N	NO_3_-N, Cl^-^, Na^+^, DO	0.8
Z_5_	3	NH_4_^+^-N	NO_3_-N, Cl^-^	0.79

Accordingly, the robust scores along the first PC1 distinctly differentiate between leachate (LW) and groundwater samples (MW) in the total dataset, while also being robust to outliers (Fig. 4B). The application of RPCA identified 110 outliers, constituting 22.9% of the total samples, which predominantly include leachate samples (LW). This suggests that the score, computed as a weighted linear combination of multivariate hydrochemical parameters, serves as an effective groundwater pollution index for assessing the impact of leachate on groundwater quality. Nevertheless, it is important to note that the eigenvectors, representing the loadings as weights of hydrochemical parameters, obtained from RPCA can be variable depending on the specific monitoring data used. This result significantly demonstrates that RPCA effectively reduces the dimensionality of compositional data and elucidates the impact of leachate contamination of groundwater by estimating a covariance structure that is robust to outlier samples. Additionally, the computation of scores involves complex transformations of the observed concentrations of multiple parameters into log-ratio values. Thus, we aim to identify critical subcompositioal parts that reflect the variability in RPCA scores and introduces their ILR-coordinate as a singular groundwater pollution index (GPI). This index serves as a versatile purpose tool for assessing the impact of leachate on groundwater quality.

### Development of ILR-based groundwater pollution index (GPI)

**Figure 5:**
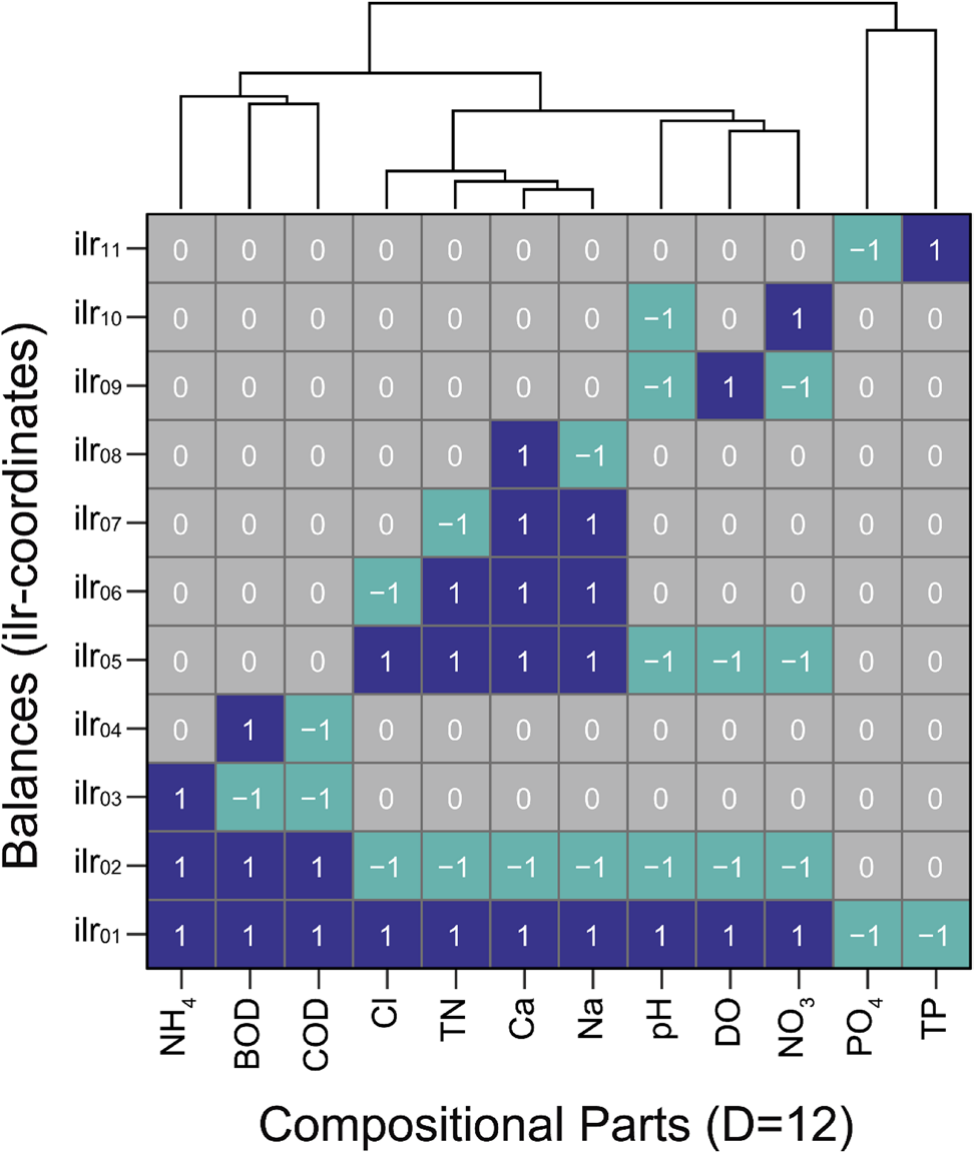
Diagram of sequential binary partition (SBP) for D = 12 compositional parts, used in partitioning the full set of hydrochemical parameters for transformation into 11 balances (ilr-coordinates). The figure shows values of 1 and − 1, representing the compositional parts assigned as the numerator and denominator, respectively, for each balance.

In the context of multivariate CoDa, although the RPCA provides useful scores for evaluating the influence of leachate on groundwater quality, this study has adopted ILR transformation to develop a more straightforward method for formulating a univariate GPI. As explained above (in Sect. 2.2.), the ILR transformation results in D-1 Cartesian coordinates, known as balances, based on an orthonormal basis established through the Sequential Binary Partition (SBP) of D selected components. Here, we construct the SBP based on the PC loadings expressed with CLR (Fig. 4), which informs about the important subcompositional parts and their relationships showing the leachate pollution in groundwater quality. Figure 5 illustrates the SBP for the case of a D = 12 subcomposition partitioning the full set of hydrochemical parameters in accordance with the results of RPCA. From this partitioning, the eleven (D-1) independent isometric log-ratio (ILR) coordinates have been derived, according to Eq. (1).

Based on the SBP, we identified the second balance (labeled as ILR2 in Fig. 5 and Z1 in Table 3), which represents a binary partition excluding total P and PO_4_^3-^, as a critical ILR-coordinate for evaluating the impact of leachate on groundwater. The selected ILR-coordinate (Z1) uses BOD, COD, and NH_4_^+^-N ions, which is mainly produced from carcass decomposition, as the numerator; meanwhile the denominator involves Na^+^, Ca^2+^, H^+^, NO_3_^-^, DO, Cl^-^ and NO_3_^-^-N ions, which are relatively dominant in the background groundwater affected by agricultural activities and oxic conditions. This log-ratio effectively retains the relative information of the data as shown in the results from RPCA exhibiting a significant correlation (ρ = 0.56) with the first principal component score (PC1) (Table 3). Additionally, it shows a positive correlation (ρ = 0.56) with electrical conductivity (EC) and a negative correlation (ρ = 0.56) with redox potential (ORP). Consequently, this ILR-coordinate is considered a reliable GPI in terms of ratio for assessing the effects of leachate on groundwater quality.

**Table 3:** Performance metrics (accuracy, sensitivity, and specificity) of the selected isometric log-ratio (ilr) based groundwater pollution index (GPI) compared with environmental criteria, suggesting the optimal cutoff value for effective groundwater pollution evaluation (positive rate).

Selected GPI (ilr)	Cutoff	Accuracy	Sensitivity	Specificity	Positive rate
Z_3_	− 0.8655	0.7762	0.67	0.88	0.37

We further examined different ILR-coordinates derived from subcompositions with a reduced number of parts (specifically, D = 7, 5, and 3 parameters), using the same procedure to develop more simplified versions of the GPI. These ILR-coordinates not only correlate well with the PC1 but also effectively account for the variations in EC and ORP (Table 3). This result suggests that the ILR-coordinates sufficiently explain the relative information relevant to the hydrochemical processes by focusing on key parameters, rather than incorporating all measured parameters. This is due to the fact that the ILR transformation ensures the principle of subcompositional coherence of compositional data^60^.

**Figure 6:**
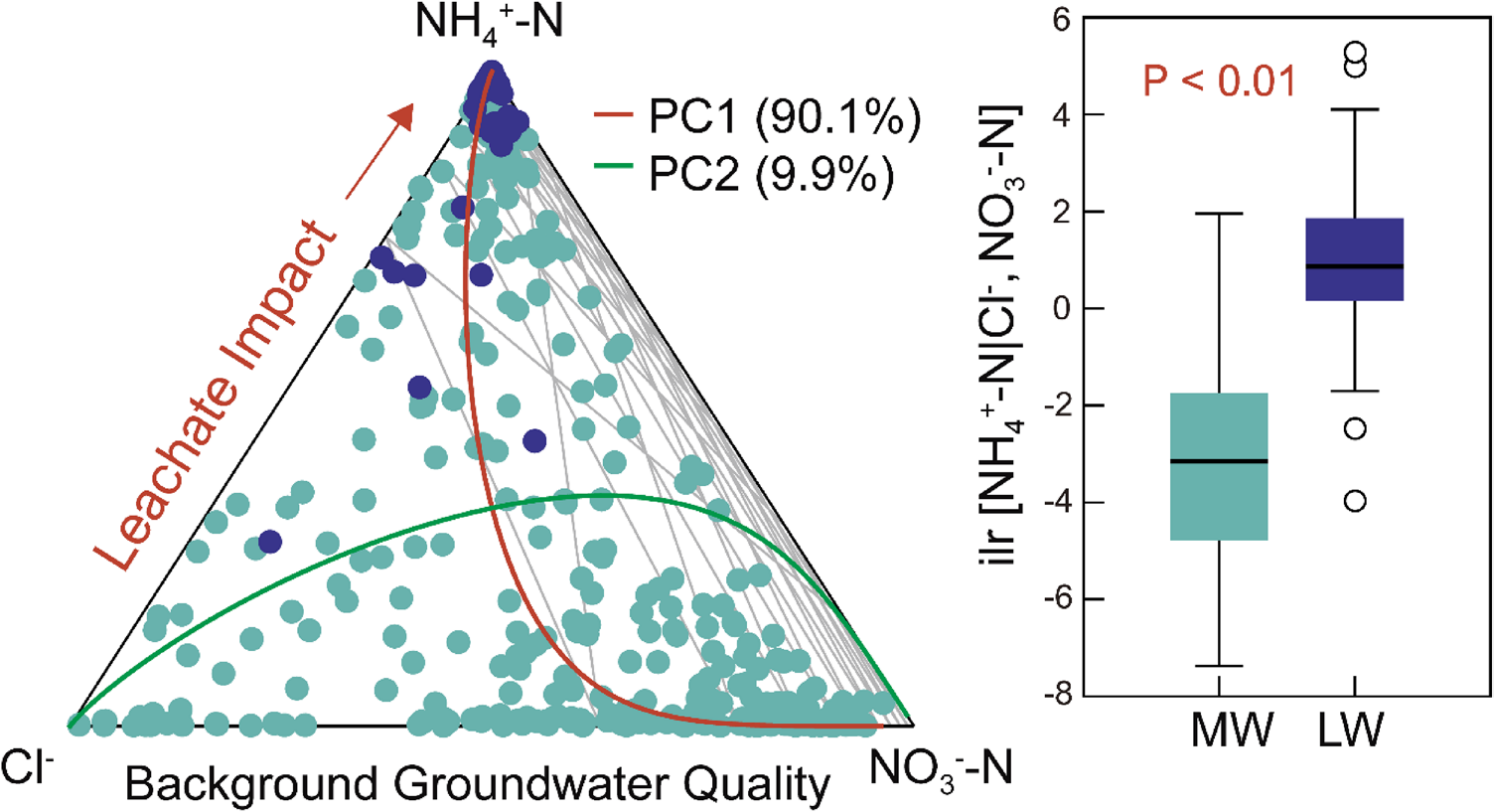
Ternary diagram illustrating the relative compositional changes among the subcompositions of NH_4_^+^-N, NO_3_^-^-N, and Cl^-^, with principal component 1 (PC1) highlighting the impact of leachate on groundwater (Left), and comparative analysis of the isometric log-ratio values for these subcompositions between Monitoring Wells (MW) and Leachate Wells (LW) (right).

**Figure 7:**
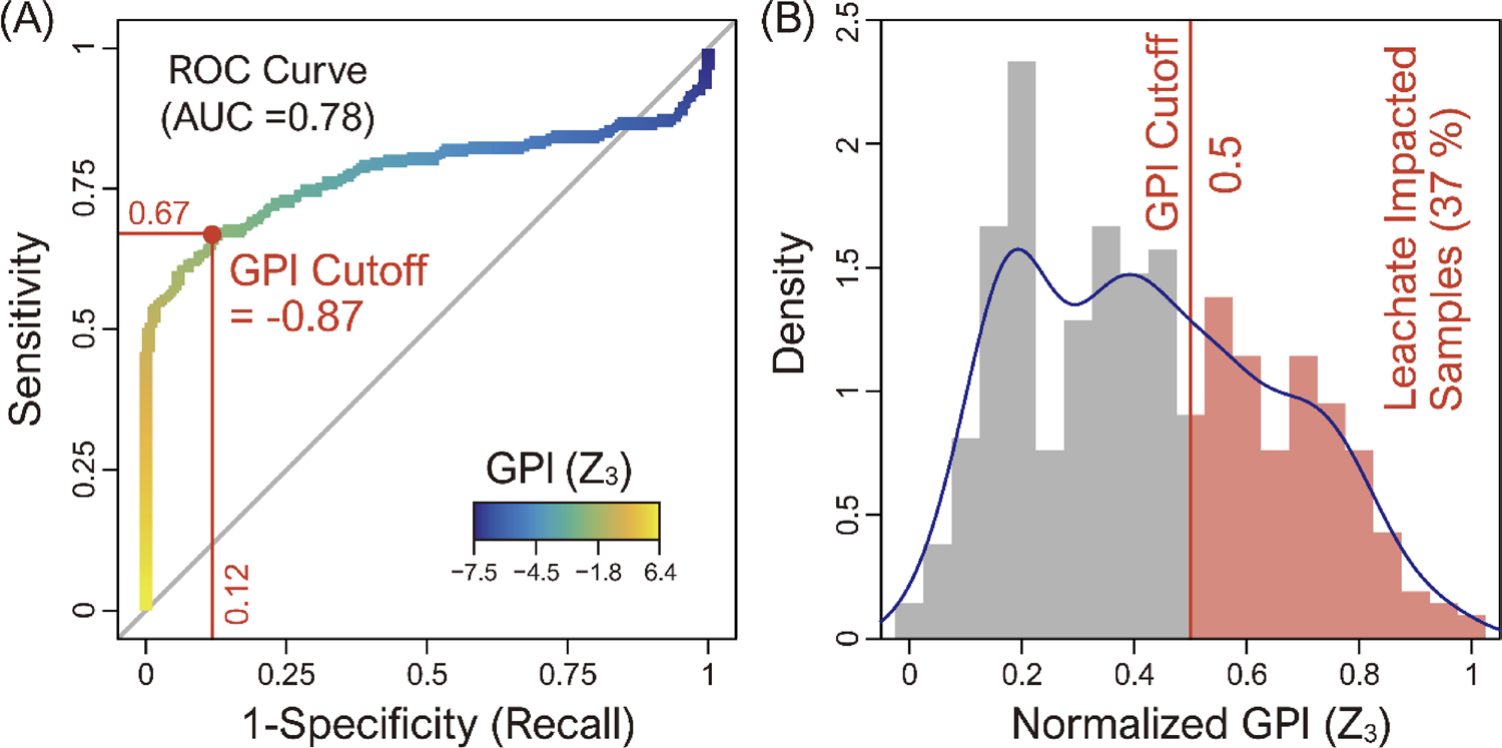
(**A**) Receiver operating characteristic (ROC) curve illustrating the classification performance of the ilr-based groundwater pollution index (GPI) in terms of sensitivity and 1-specificity, compared against environmental criteria (ME, 2011), and (**B**) histogram depicting the distribution of normalized ilr-based GPI values for the entire dataset of groundwater samples (n = 420), highlighting that 37% of the samples were identified as leachate-impacted using the optimal cutoff value of 0.5 (Z3 = − 0.87).

The ternary diagram in Fig. 6 shows the distribution of three subcompositional parts (NH_4_^+^-N, Cl^-^ and NO_3_^-^-N) characterized by two ILR-coordinates (ILR[NH_4_^+^-N |Cl^-^, NO_3_^-^-N] and ILR[Cl^-^| NO_3_^-^-N]) in the Euclidean space. The first of these coordinates corresponds to the Z3 in Table 3 explaining 90.1% of the total variance in the distribution. This ratio reflects the increase in NH_4_^+^-N relative to Cl^-^ and NO_3_^-^-N, and differs shows a significant difference (*p* < 0.05) between leachate (MW) and groundwater (GW) (Fig. 7B). Consequently, the ILR-coordinates of three specifically selected parts (NH_4_^+^-N, Cl^-^ and NO_3_^-^-N) provide the most simplified and practical form of GPI while optimally maintaining the essential information of groundwater quality monitoring data. We propose a univariate GPI to quantify the impact of leachate on groundwater, using the following ILR equation:

5 $$ GPI\left({Z}_{3}\right)=\sqrt{\frac{2}{3}} ln\frac{[{NH}_{4}^{+}-N]}{\sqrt{{NO}_{3}^{-}-N][{Cl}^{-}]}}$$

The ILR-coordinate (Z3), proposed as a GPI, was compared with the assessment results of leachate impact on groundwater, as outlined by the government's environmental criteria (mentioned in Sect. 2.1.). For this, data samples were categorized into binary groups based on varying ILR values, and these classifications were then juxtaposed with those designated as contaminated or uncontaminated according to the environmental criteria, measuring sensitivity and specificity. Such a comparison not only validates the GPI's potential as a viable alternative to the environmental criteria but also suggests an appropriate GPI cutoff that aligns with the criteria.

We determined the optimal cutoff for the GPI using a receiver operating characteristic (ROC) curve with an area under curve (AUC) of 0.78, which graphically represents sensitivity versus 1-specificity (recall) across various cutoff points. The optimal cutoff, determined at the point where sensitivity is maximized and 1-specificity is minimized, was identified as -0.87 (as shown in Fig. 7A). At this point, the sensitivity was 0.67, correctly identifying 67% of samples as contaminated according to the Environmental Criteria, while the specificity was 0.88, accurately classifying 88% of uncontaminated samples (Table 3). These results validate the effectiveness of GPI in differentiating between contaminated and uncontaminated groundwater, confirming its reliability as a tool for environmental pollution assessment. Finally, the ILR-based GPI was adjusted to center around the cutoff and normalized between 0 and 1, utilizing the maximum and minimum values according to Eq. (4). Within this normalized scale, a GPI value exceeding 0.5 is established as the threshold for identifying leachate contamination, in accordance with the government's environmental criteria. Notably, this normalized GPI revealed that more than 80% of the entire monitoring dataset exceeded this 0.5 threshold, suggesting significant contamination.

This study utilized groundwater monitoring data from areas affected by the 2010–2011 foot-and-mouth disease outbreak in South Korea to highlight the effectiveness of CoDa in distinguishing between leachate contaminated and uncontaminated groundwater. The GPI, developed using CoDa and RPCA, significantly improves the accuracy and reliability of assessments by considering the relative nature of hydrochemical data, which is often overlooked by traditional statistical methods. The proposed GPI was validated against government environmental standards, demonstrating high sensitivity and specificity in distinguishing between contaminated and uncontaminated groundwater. These results not only validate the reliability of the GPI as an environmental pollution assessment tool but also suggest that it can play a crucial role in complementing existing environmental standards to enhance groundwater resource monitoring and management. Specifically, the CoDa approach proposed in this study overcomes the limitations of traditional methods by considering the relative nature of hydrochemical data, thereby providing a more accurate and reliable assessment tool. This is vital for policy making and environmental management, contributing to the protection and sustainable management of groundwater resources. Furthermore, the methodology and results of this study offer essential groundwork for future research and policy development.

## Summary and conclusion

This research introduces an innovative Groundwater Pollution Index (GPI) that employs compositional data analysis (CoDa) and robust principal component analysis (RPCA) to advance the assessment of groundwater quality. Utilizing data collected from the groundwater monitoring of sites affected by the 2010–2011 foot-and-mouth disease outbreak in South Korea, this study highlights the effectiveness of CoDa in distinguishing significant hydrochemical differences between leachate-influenced groundwater and unaffected background samples.

The GPI is meticulously developed through a process that involves selecting essential subcompositional parts, specifically NH_4_^+^-N, Cl^-^ and NO_3_^-^-N, using RPCA, conducting isometric log-ratio (ILR) transformation to address the compositional nature of hydrochemical data, and normalizing these results in accordance with environmental standards. The validation of the GPI against established government criteria, supported by receiver operating characteristic (ROC) curve analysis with an area under curve (AUC) of 0.78 underscores its potential as a robust alternative tool for groundwater pollution assessment. With a sensitivity of 0.67 and specificity of 0.88, the GPI effectively distinguished between contaminated and uncontaminated groundwater samples.

A significant contribution of this study is the emphasis on the importance of CoDa, particularly the ILR transformation, in overcoming the methodological limitations (i.e., outlier and data closure) of traditional statistical methods that often overlook the relative nature of hydrochemical data. This approach significantly enhances the accuracy and reliability of groundwater quality assessments. The proposed GPI aligns with existing environmental standards while serving as a more precise and reliable assessment tool, providing a robust framework for effective monitoring and management of groundwater resources. This is crucial for policy decision-making and environmental management, contributing to the protection and sustainable management of groundwater resources. Furthermore, the methodology and results of this study provide essential groundwork for future research and policy development. Researchers can build upon this work to conduct new studies and further refine the GPI, advancing the field of groundwater quality assessment.

## Data Availability

The datasets used and/or analysed during the current study available from the corresponding author on reasonable request.
