# Author Correction: Using isometric log-ratio in compositional data analysis for developing a groundwater pollution index

**DOI:** 10.1038/s41598-025-90356-x

**Published:** 2025-02-19

**Authors:** Junseop Oh, Kyoung-Ho Kim, Ho-Rim Kim, Sunhwa Park, Seong-Taek Yun

**Affiliations:** 1 Department of Earth and Environmental Sciences, Korea University, Seoul, 02841 South Korea; 2 Korea Environment Institute, Sejong, 30147 South Korea; 3 Korea Institute of Geoscience and Mineral Resources, Daejeon, 34132 South Korea; 4 National Institute of Environmental Research, Incheon, 22689 South Korea

Correction to: *Scientific Reports* 10.1038/s41598-024-63178-6, published online 28 May 2024

The Acknowledgements section in the original version of this Article was incomplete.

This study was financially supported by the 2012 project (Title: A Study on Groundwater Quality Management Measures around Livestock Burial Sites (I); NEIR-2021-04-02-058) funded by the National Institute of Environmental Research (NEIR) and the Ministry of Environment of South Korea. The completion of this work was supported by the Korea University Grant and the Research Project (Development of integrated decision support model for environmental impact assessment project: 2022-003R) of Korea Environment Institute (KEI). Partial support was also given by the Basic Research Project (GP2021-007) of the Korea Institute of Geoscience and Mineral Resources (KIGAM).

now reads:

This study was financially supported by the 2012 project titled A Study on Groundwater Quality Management Measures around Livestock Burial Sites (I) (NEIR-2021-04-02-058), funded by the National Institute of Environmental Research (NEIR) and the Ministry of Environment of South Korea. Furthermore, this research was supported by the Research Project Development of integrated decision support model for environmental impact assessment project (2022-003R), conducted by the Korea Environment Institute (KEI) and funded by the research and development project (Project No. 2020002990007) of the Environmental Industry & Technology Institute (KEITI) and the Ministry of Environment (MOE).

Additionally, partial support was provided by the Korea University Grant and the Basic Research Project (GP2021-007) of the Korea Institute of Geoscience and Mineral Resources (KIGAM).

The original Article has been corrected.

